# Long Term Adaptation to Heat Stress: Shifts in the Minimum Mortality Temperature in the Netherlands

**DOI:** 10.3389/fphys.2020.00225

**Published:** 2020-03-18

**Authors:** Mireille A. Folkerts, Peter Bröde, W. J. Wouter Botzen, Mike L. Martinius, Nicola Gerrett, Carel N. Harmsen, Hein A. M. Daanen

**Affiliations:** ^1^Department of Human Movement Sciences, Faculty of Behavioural and Movement Sciences, Vrije Universiteit Amsterdam, Amsterdam Movement Sciences, Amsterdam, Netherlands; ^2^Leibniz Research Centre for Working Environment and Human Factors (IfADo), Dortmund, Germany; ^3^Institute for Environmental Studies (IVM), Vrije Universiteit Amsterdam, Amsterdam, Netherlands; ^4^Statistics Netherlands, Voorburg, Netherlands

**Keywords:** mortality, temperature, climate change, human adaptation, older adults, minimum mortality temperature

## Abstract

It is essentially unknown how humans adapt or will adapt to heat stress caused by climate change over a long-term interval. A possible indicator of adaptation may be the minimum mortality temperature (MMT), which is defined as the mean daily temperature at which the lowest mortality occurs. Another possible indicator may be the heat sensitivity, i.e., the percentage change in mortality per 1°C above the MMT threshold, or heat attributable fraction (AF), i.e., the percentage relative excess mortality above MMT. We estimated MMT and heat sensitivity/AF over a period of 23 years for older adults (≥65 years) in the Netherlands using three commonly used methods. These methods are segmented Poisson regression (SEG), constrained segmented distributed lag models (CSDL), and distributed lag non-linear models (DLNM). The mean ambient temperature increased by 0.03°C/year over the 23 year period. The calculated mean MMT over the 23-year period differed considerably between methods [16.4 ± 1.2°C (SE) (SEG), 18.9 ± 0.5°C (CSDL), and 15.3 ± 0.4°C DLNM]. MMT increased during the observed period according to CSDL (0.11 ± 0.05°C/year) and DLNM (0.15 ± 0.02°C/year), but not with SEG. The heat sensitivity, however, decreased for the latter method (0.06%/°C/year) and did not change for CSDL. Heat AF was calculated for the DLNM method and decreased with 0.07%/year. Based on these results we conclude that the susceptibility of humans to heat decreases over time, regardless which method was used, because human adaptation is shown by either an increase in MMT (CSDL and DLNM) or a decrease in heat sensitivity for unchanged MMT (SEG). Future studies should focus on what factors (e.g., physiological, behavioral, technological, or infrastructural adaptations) influence human adaptation the most, so it can be promoted through adaptation policies. Furthermore, future studies should keep in mind that the employed method influences the calculated MMT, which hampers comparability between studies.

## Introduction

Humans possess a great capacity to acclimatize to heat (Periard et al., 2016). Over a period of approximately 10 days, cardiovascular, thermoregulatory and fluid control mechanisms are optimized so that heat strain has a reduced effect on human well-being and performance. These acute adaptations are well documented (Périard et al., 2015; Periard et al., 2016), but adaptations to long term exposure (i.e., several years) are essentially unknown. This is problematic for accurate estimations of future morbidity and mortality in the face of climate change, with numerous scientific papers making a disclaimer for the unknown effects of the “human adaptation” (Sanderson et al., 2017). When adaptation to heat is assumed, it has a considerable impact on predicted mortality and associated societal costs (Díaz et al., 2019).

Heat related excess mortality is mainly observed in elderly subjects (Baccini et al., 2008). Older adults (>65 years) are most at risk for temperature related mortality due to intrinsic changes in the thermoregulatory system, like a reduced sweat response and thirst sensation (Foster et al., 1976; Kenney and Chiu, 2001). In addition, older adults are often less physically fit and have more illnesses and disabilities what makes them also more susceptible to heat-related morbidity and mortality (Koppe et al., 2004). However, older adults are able to acclimatize to the heat (Inoue et al., 1999; Best et al., 2014) when a sufficient number of days for adaptation is allowed (Daanen and Herweijer, 2015). Furthermore, they may be more resilient to heat in hot cities than in colder cities (Worfolk, 2000). This increased resilience may be due to better housing, behavioral adaptations, increased use of air conditioners (JRAIA, 2018), improved awareness of heat impact due to public campaigns, but also due physiological adaptations of the human body to the heat.

Mortality data, especially in the older population, often exhibit a U- or V-shaped relationship with temperature (Kunst et al., 1993), with the number of deaths increasing for temperatures below or above the so-called minimum mortality temperature (MMT). MMT is the mean daily temperature at which the lowest mortality occurs and quantifies the threshold between the cold and heat mortality slope. The term MMT was first used to illustrate the considerable differences in the temperature-mortality relationship in the United States (Curriero et al., 2002): MMT for Boston was 21°C and 27°C for Miami. Cities show higher MMT values when located closer to the equator (Hajat and Kosatky, 2010). This is observed for European cities (Baccini et al., 2008), but also within countries. Tobias et al. (2017), for instance, showed that hotter cities have a higher MMT in Spain.

Recent studies have proposed the use of the MMT as a potential indicator of human long-term adaptation to heat in case the MMT shifts to a higher temperature (Barrett, 2015; Todd and Valleron, 2015; Åström et al., 2016). If humans become less susceptible to heat, an increase in MMT can be expected over time, similar to higher MMT values in warmer cities due to geographic differences. Todd and Valleron (2015) found an increase in MMT from 17.5°C in 1968–1981 to 17.8°C in 1982–1995 and 18.2°C in 1996–2009 in France. An increase in ambient temperature of 1.6°C over these years was accompanied by an increase in MMT of 0.8°C. For comparison: a temperature difference of 1.6°C between geographical areas was accompanied by a 1.1°C increase in MMT.

There are several approaches to calculate MMT from temperature-mortality time-series data and different methods have been used in the studies mentioned above. One simple statistical model predicting the logarithm of the death counts by actual temperature is the segmented Poisson regression model (SEG) providing estimates of the breakpoint (MMT) as well as of the negative temperature slope in the cold and positive slope in the heat, while accounting for covariates, e.g., day of week (Muggeo, 2003, 2008a, 2017). Only focusing on the temperature influence on the same day, SEG neglects the time series structure, and especially does not consider lagged effects of temperature on mortality. However, it is easily applicable to separate one-year periods allowing for assessing the development of MMT over the whole observation period, as well as of heat and cold sensitivity from the respective slopes. The constrained-segmented distributed lag model (CSDL) also includes MMT as estimated parameter, but extends the simple V-shape model by considering non-linear lagged effects as well as long-term and seasonal trends in the time series (Muggeo, 2008b, 2010). By additionally relaxing the linear V-shape assumption, so-called distributed lag non-linear models (DLNM) allow to fit more flexible temperature-mortality relationships to such time series data (Gasparrini, 2011; Gasparrini et al., 2017). DLNM requires an extra step applying a search algorithm for finding the MMT (Tobias et al., 2017). Both the CSDL and DLNM models usually rely on longer observation periods covering at least 10 – 20 years. Thus, in order to assess the development of MMT over time, recent studies fitted the data to the observations from non-overlapping (Carson et al., 2006; Petkova et al., 2014; Todd and Valleron, 2015) or partly overlapping (Åström et al., 2016; Chung et al., 2017) sub-periods.

It is unknown if the variation in MMT is due to human adaptation or due to the methods used to calculate MMT as outlined above. Therefore, the aim of the current study was twofold: we investigated changes in MMT in the Netherlands over a period of 23 years, from 1995 to 2017, for older adults (≥65 years), whilst comparing the three previously mentioned models (SEG, CSDL, and DLNM).

## Materials and Methods

### Database

The daily number of deaths and population size in the Netherlands, obtained from Statistics the Netherlands (CBS), and temperature data, obtained from the Royal the Netherlands Meteorological Institute (KNMI) from January 01, 1995 to December 31, 2017 were used for the calculations in this study. Only mortality in the age group of 65 years and older was processed, because this group is reportedly the most vulnerable to extreme ambient temperatures (Koppe et al., 2004).

Hourly ambient temperature was obtained from five weather stations representative for the Netherlands: Station De Bilt (in the center of the Netherlands), Station Eelde (rural area, farmland, northern part of the Netherlands), Maastricht (average sized city, southern part of the Netherlands), Rotterdam (large city near the coast, western part of the Netherlands), and Schiphol airport (industrial area, amid densely populated areas, western part of the Netherlands). Daily temperature used in this study was obtained by averaging the hourly values over the five weather stations and time.

### Model Calculations

Calculations were performed using R version 3.6.1 (R Core Team, 2019). For the entire time series, a segmented Poisson regression model (SEG) allowing for over dispersion (Wood, 2006) was fitted to daily mortality with daily mean temperature as predictor and day-of-week as only covariate using the R package *segmented* (Muggeo, 2003, 2008a, 2017). Estimates and SE were provided for MMT and for the cold and heat slope parameters. Relative risks (RR) with mortality at MMT as reference were calculated by exponentiation of the slope parameters multiplied with the difference of MMT to temperature. Sensitivities to cold and heat calculated by exponentiation of the slope estimates were expressed as percentage change per degree decrease or increase in temperature from MMT, respectively.

Similarly, we also obtained MMT and sensitivities to heat and cold by fitting constrained segmented distributed lag (CSDL) models using the package *modTempEff* (Muggeo, 2008b, 2010) controlling for the day-of-week, while considering lag temperature effects and adjusting for annual and seasonal trends with spline functions as suggested by the software manual (Muggeo, 2010).

Distributed lag non-linear models (DLNM) were fitted using the *dlnm* package (Gasparrini, 2011), also including day-of-week covariate while considering lag temperature effects up to 25 days and adjusting for long-term and seasonal trends with spline functions using eight degrees-of-freedom per year. MMT with SE was estimated by a search algorithm over the fitted response function (Tobias et al., 2017). The sensitivities to heat and cold were calculated using the attributable fraction (AF), expressed as percentage relative excess mortality integrated over the lag periods and temperatures above and below MMT, respectively (Gasparrini and Leone, 2014).

### Shift in MMT and Sensitivity to Heat and Cold

The shift in MMT over time in the SEG method was calculated by fitting the SEG model for every year separately. For CSDL and DLNM, the models were repeatedly fitted to reduced series from a sliding 15-year window, which was shifted by 1-year increments throughout the entire observation period.

A shift of MMT and heat and cold sensitivities (AF for DLNM) was assessed by performing linear regression analyses for changes in the parameters weighted by their inversed SE over time, while statistical significance was assumed for *p*-values < 0.05.

## Results

The average number of deaths in the Netherlands over the investigated 23-year time span was 382 ± 40 (SE) individuals per day. The mean daily temperature in the Netherlands was 10.5 ± 6.3°C. The mean temperature increase over the observed period was 0.03°C/year. The number of people over 65 years increased from 2.0 million to 3.2 million between 1995 and 2017, but their mean age was stable at 74.5 ± 0.1 years over the investigated period (CBS, 2019a).

**FIGURE 1:**
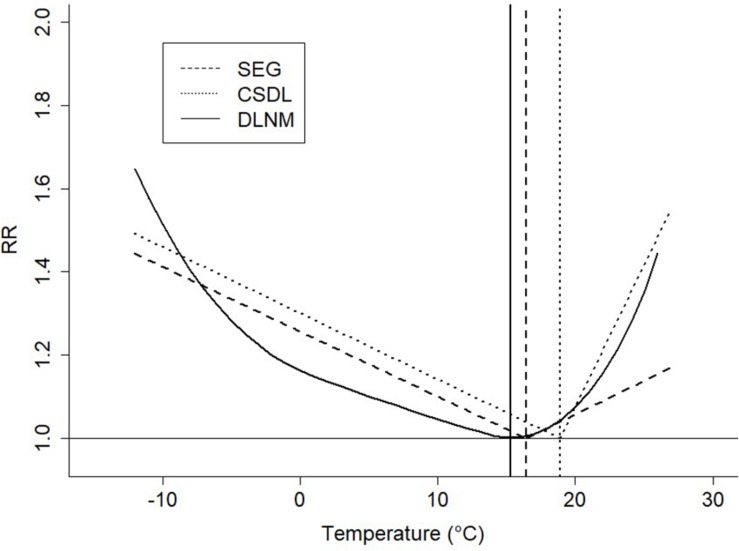
Temperature related to the relative risk (RR) for mortality of older adults (≥65 years) for three different methods: Segmented Poisson regression (SEG), constrained segmented distributed lag models (CSDL) and distributed lag non-linear models (DLNM), during the 23-year period from January 01, 1995 to December 31, 2017 in the Netherlands. The minimum mortality temperature (MMT) estimated by the three different methods is shown with the vertical lines. The slopes of the lines represent the cold/heat sensitivity of the SEG and CSDL method, whereas the cold/heat attributable fraction (AF) of the DLNM method is determined as relative excess mortality integrated over the temperatures above and below MMT, respectively.

Figure 1 shows the relative risk (RR) of mortality at different daily mean temperatures over the entire 23-year period according to the three different methods. The SEG and CSDL methods assume a linear relation and, therefore, have a V-shaped estimation of the temperature-mortality curve. The DLNM method assumes a non-linear relation and, therefore, has a more U-shaped curve. The mean calculated MMT, and cold and heat sensitivity/AF are shown in Table 1. Large differences are shown for the calculated MMT between methods with values between 15.3 ± 0.4°C for the DLNM method and 18.9 ± 0.5°C for the CSDL method. Cold sensitivity was similar with 1.3 ± 0.2% and 1.3 ± 0.3% for, respectively, the CSDL and SEG methods, but a large difference was found for the heat sensitivity with 5.6 ± 0.6% for the CSDL method and 1.5 ± 0.8% for the SEG method. The AF to the cold calculated with the DLNM method was higher than the AF to the heat, with, respectively, 5.0 ± 0.3% and 1.1 ± 0.2%.

**TABLE 1:** The minimum mortality temperature (MMT) (Mean ± SD), cold and heat sensitivity (SEG and CSDL models) and attributable fraction (AF) (DLNM model) (%) calculated for the 23-year period from January 01, 1995 to December 31, 2017 in the Netherlands with the three different methods: Segmented Poisson regression (SEG), constrained segmented distributed lag (CSDL) model, and distributed lag non-linear models (DLNM).

Method	MMT (°C)	Cold sensitivity (%/°C)/AF (%)	Heat sensitivity (%/°C)/AF (%)
SEG	16.4 ± 1.2	1.3 ± 0.3^a^	1.5 ± 0.8^a^
CSDL	18.9 ± 0.5	1.3 ± 0.2^a^	5.6 ± 0.6^a^
DLNM	15.3 ± 0.4	5.0 ± 0.3^b^	1.1 ± 0.2^b^

*^a^Sensitivity. ^b^AF.*

**FIGURE 2:**
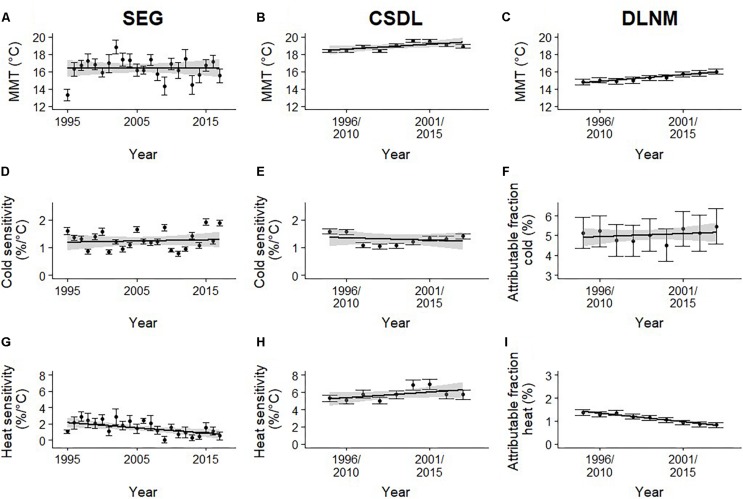
Changes in minimum mortality temperature (MMT) **(A–C)**, cold sensitivity/attributable fraction (AF) **(D–F)**, and heat sensitivity/AF **(G–I)** with standard errors and gray shaded 95% confidence bands estimated by three different methods: Segmented Poisson regression (SEG) (left panel), constrained segmented distributed lag model (CSDL) (middle panel), and distributed lag non-linear models (DLNM) (right panel) of daily death counts for older adults (≥65 years) related to daily mean temperature for the 23-year period from January 01, 1995 to December 31, 2017 in the Netherlands.

Figure 2 shows the calculated MMT per year for the SEG method and with a sliding 15-year window for the CSDL and DLNM methods. Cold and heat sensitivity are reported for the SEG and CSDL methods and cold and heat AF for the DLNM method. A significant increase in MMT of 0.11 ± 0.05°C and 0.15 ± 0.02°C per year was observed for the CSDL (*p* < 0.001) and DLNM (*p* < 0.05) methods, respectively. However, no significant increase in MMT was found with the SEG method (*p* = 0.96). Cold sensitivity did not change over time in both the CSDL (*p* = 0.57) and the SEG methods (*p* = 0.69). Heat sensitivity did not change significantly in the CSDL method (*p* = 0.12), but did decrease significantly with the SEG method (*p* = 0.01) with 0.06%/°C/year. No significant difference in cold AF is shown (*p* = 0.511), however, there was a significant decrease of 0.07%/year in heat AF (*p* < 0.001).

## Discussion

The aim of the current study was to investigate the change in MMT, and cold and heat sensitivity/AF over a 23-year period for older adults (≥65 years) in the Netherlands using three different methods (SEG, CSDL, and DLNM). Furthermore, the differences between the three methods were also analyzed to investigate the influence of the employed method on the results.

The calculated mean MMT and heat sensitivity over the 23-year period differed considerably between methods. The CSDL method resulted in the highest MMT (18.9 ± 0.45°C) and heat sensitivity (5.6 ± 0.6%). The high heat sensitivity is a result of the higher MMT, as only days with a higher temperature than the MMT are included in the calculation of the heat sensitivity. In other words, the data is refined to the steepest part of the mortality curve (Figure 1) resulting in a high heat sensitivity. The SEG method has a lower MMT (16.4 ± 1.2°C) and thus mortality data is included of more moderate temperatures above this low MMT threshold. The MMT calculated with the DLNM method was the lowest of all three methods with 15.3 ± 0.4°C. The cold AF is higher than the heat AF with 5.0 ± 0.3% and 1.1 ± 0.2%, respectively, which can be explained with the fact that the average daily temperature in the Netherlands (10.5 ± 6.3°C) is below MMT and thus cold days are more prominent in the Netherlands. The differences in calculated MMT between methods are most likely due to the way the MMT is calculated in the models. Both the CSDL and DLNM method control for the day-of-week, annual and seasonal trends and consider lag temperature, while this is not the case in the SEG method. In addition, the DLNM method uses a non-linear approach, while both the SEG and CSDL method are linear. These results show that the used method has a large effect on the calculated MMT and the accompanying cold and heat sensitivity/AF. Comparability between studies employing different methods is therefore hampered.

The results of all three methods indicate that the susceptibility to heat in the Netherlands is declining over time. Two of the three methods (DLNM and CSDL) show an increase in MMT for adults of 65 years and older over the 23-year period (see Figures 2A–C). The SEG method does not show an increase in MMT, but does show a decrease in heat sensitivity over time from about 2% to 1% per degree Celsius. This indicates that less people die at similar heat exposure suggesting a gradual adaptation to heat. The CSDL method shows a slight increase in heat sensitivity, although not significant. This has to be considered in relation with the increasing MMT as the dataset for heat sensitivity will contain less moderate temperature days and increasingly more hot days. Therefore, it does not mean people are getting more susceptible to the heat based on the CSDL method. The same explanation accounts for the decrease in heat AF calculated with the DLNM method. As the MMT increases over time there are less days with a mean ambient temperature higher than the MMT and therefore less deaths are attributed to the heat. Cold sensitivity and AF does not change over the years for stable MMT (SEG) or for increasing MMT (CSDL and DLNM).

The observed increase of the MMT from 0.11 to 0.15°C/year, accompanied with mean daily temperature increases of about 0.03°C/year, is in line with previous studies (Todd and Valleron, 2015; Åström et al., 2016; Chung et al., 2018). In France the observed shift in MMT was lower than in the current study with 0.025°C/year for adults over 65 years old and an increase in summer temperature of 0.057°C/year (Todd and Valleron, 2015). In Sweden and Japan the shift in MMT was more comparable with the current study with, respectively, 0.08°C/year and about 0.12°C/year for the whole population (Åström et al., 2016; Chung et al., 2018). In Sweden the mean ambient temperature increased with 0.018°C/year over the observed period and in the study of Chung et al. (2018) the increase in mean ambient temperature was not reported. In the study of Todd and Valleron (2015) Generalized Additive Models were used and in the studies of Åström et al. (2016) and Chung et al. (2018) the DLNM method was used similar to our study. The difference in applied methods may explain the smaller observed shift in MMT reported for France. However, all studies, including the current study for the Netherlands, suggest human adaptation to climate change.

These human adaptations to the increasing ambient temperatures can be attributed to multiple factors, such as physiological, behavioral, technological adaptations or changes in infrastructure (Hondula et al., 2015). Repeated heat exposures lead to physiological adaptations in heart rate, body core temperature and sweat rate that slowly decay (Daanen et al., 2018), and thus may lead to a more or less permanent state of heat acclimation (Casadio et al., 2016). In line with this, it has been shown that mortality is considerably higher in the heat waves early in summer when compared to successive heat waves, probably partly due to heat acclimation in the subjects that survived the initial heat waves (Kysely and Kriz, 2008). Further, people born and raised in warm areas show reduced excess mortality in the heat when moved to relatively cold areas (Vigotti et al., 2006). Behavioral changes may occur because people become more aware of the impact of high ambient temperatures and raised awareness from the government. For example, since 2007 in the Netherlands, a heat health warning systems (HHWS) is activated if there is a high chance of five consecutive days with an ambient temperature exceeding 27°C (Lowe et al., 2011). The aim of the HHWS is to warn people when extremely high temperatures are expected and to give behavioral recommendations (e.g., drink more, reduce physical activity) during these days. Technological and infrastructural changes over the years include improved building insulation that reduces heat loss in the cold and prevents heating of the house in hot periods. Air conditioning is an effective way of reducing heat strain. In the Netherlands the air conditioner demand already increased with about 24% between 2012 and 2017 according to the Japan Refrigeration and Air Conditioning Industry Association (JRAIA, 2018), probably contributing to the observed reduction in heat susceptibility over the years. A long term adaptation to climate change observed in endotherms is an increase in the body surface to mass ratio to enhance heat loss (Gardner et al., 2011). In humans the body surface to mass ratio is higher in tropical than in cold areas (Katzmarzyk and Leonard, 1998). However, the body surface to mass ratio of the Dutch population shows a consistent linear decline over the investigated period (CBS, 2019b), so no signs of climate change related morphological changes are observed.

It has to be noted that the decreased susceptibility to heat over time may not only be related to climate change as suggested by Todd and Valleron (2015). Arbuthnott et al. (2016) showed that decreased heat susceptibility is a process that is not only visible in the last decades, but already started a century ago, when climate change was still negligible. Ten out of eleven included papers in their study found some evidence of decreasing susceptibility for heat over time. Cold susceptibility changes were negligible. It was argued that both planned adaptive measures, such as HHWS and improved buildings as well as adaptive behavior, improved health and treatment of heat casualties could explain the changes. Still, climate change may accelerate the adaptations as Todd and Valleron (2015) indicated.

Kinney (2018) argued that human adaptation should be better quantified and included in methods used for predicting the effects of climate change on human survival. With ongoing climate change and associated adaptive processes, the temperature-mortality relationships on both sides of MMT may change, with the magnitude and direction of the change being uncertain. Future studies should focus on what particular factors, like the physiological, behavioral, technological or infrastructural changes mentioned before, are influencing the reduced susceptibility to the heat the most. Once the most effective factors are identified adaptation policies may be proposed accordingly.

In this study for the CSDL and DLNM method a 15-year sliding window was chosen, which covers quite an extensive part of the in total 23 years. Using sliding windows with fewer years resulted in larger volatility accompanied with a greater standard error, indicating a less precise MMT. However, previous studies like Åström et al. (2016) used a large sliding window as well of 30 years, although we are aware that they used a much larger total time period covering more than 100 years. Furthermore, data from the KNMI shows that an increase in ambient temperature is mostly present in the previous three decades, making it less relevant to use a dataset containing a longer time period than used in the current study (KNMI, 2018).

## Conclusion

The susceptibility of humans to the heat decreases over time in the Netherlands, regardless which method was used, as human adaptation was shown by either an increase in MMT (CSDL and DLNM) or a decrease in heat sensitivity for unchanged MMT (SEG). Underlying factors for the reduced heat susceptibility may be due to physiological, behavioral, technological or infrastructural adaptations. Future studies should focus on what factor influences the human adaptation the most, so it can be promoted through adaptation policies. Further, future studies should keep into mind that the employed method influences the calculated MMT and, therefore, reduces comparability between studies using different methods.

## Data Availability Statement

The datasets generated for this study are available on request to the corresponding author.

## Author Contributions

HD, MF, WB, and MM devised the study. HD, MF, and PB designed and conceived the analyses. CH, MF, and MM collected and organized the data. MF and PB performed the statistical analysis. MF and HD wrote the first draft. All authors interpreted the data, and, after critically reviewing and providing significant editing of its content, approved the final manuscript.

## Conflict of Interest

The authors declare that the research was conducted in the absence of any commercial or financial relationships that could be construed as a potential conflict of interest.
